# Improved first trimester maternal iodine status with preconception supplementation: The Women First Trial

**DOI:** 10.1111/mcn.13204

**Published:** 2021-05-25

**Authors:** Amy E. Young, Jennifer F. Kemp, Charis Uhlson, Jamie L. Westcott, Sumera A. Ali, Sarah Saleem, Ana Garcès, Lester Figueroa, Manjunath S. Somannavar, Shivaprasad S. Goudar, K. Michael Hambidge, Audrey E. Hendricks, Nancy F. Krebs, the Women First Preconception Maternal Nutrition Trial Group

**Affiliations:** ^1^ Department of Pediatrics, Section of Nutrition University of Colorado School of Medicine Aurora Colorado USA; ^2^ Department of Community Health Sciences Aga Khan University Karachi Pakistan; ^3^ Maternal Infant Health Center Instituto de Nutrición de Centro América y Panamá (INCAP) Guatemala City Guatemala; ^4^ Women's and Children's Health Research Unit KLE Academy of Higher Education & Research's JN Medical College Belagavi India; ^5^ Department of Mathematical & Statistical Sciences University of Colorado Denver Denver Colorado USA

**Keywords:** birth length, iodine supplementation, pregnancy, salt iodization, small‐quantity lipid‐based nutrient supplement (SQ‐LNS), urinary iodine concentration (UIC)

## Abstract

Maternal iodine (I) status is critical in embryonic and foetal development. We examined the effect of preconception iodine supplementation on maternal iodine status and on birth outcomes. Non‐pregnant women in Guatemala, India and Pakistan (*n* ~ 100 per arm per site) were randomized ≥ 3 months prior to conception to one of three intervention arms: a multimicronutrient‐fortified lipid‐based nutrient supplement containing 250‐μg I per day started immediately after randomization (Arm 1), the same supplement started at ~12 weeks gestation (Arm 2) and no intervention supplement (Arm 3). Urinary I (μg/L) to creatinine (mg/dl) ratios (I/Cr) were determined at 12 weeks for Arm 1 versus Arm 2 (before supplement started) and 34 weeks for all arms. Generalized linear models were used to assess the relationship of I/Cr with arm and with newborn anthropometry. At 12 weeks gestation, adjusted mean I/Cr (μg/g) for all sites combined was significantly higher for Arm 1 versus Arm 2: (203 [95% CI: 189, 217] vs. 163 [95% CI: 152, 175], *p* < 0.0001). Overall adjusted prevalence of I/Cr < 150 μg/g was also lower in Arm 1 versus Arm 2: 32% (95% CI: 26%, 38%) versus 43% (95% CI: 37%, 49%) (*p* = 0.0052). At 34 weeks, adjusted mean I/Cr for Arm 1 (235, 95% CI: 220, 252) and Arm 2 (254, 95% CI: 238, 272) did not differ significantly but were significantly higher than Arm 3 (200, 95% CI: 184, 218) (*p* < 0.0001). Nominally significant positive associations were observed between I/Cr at 12 weeks and birth length and head circumference *z*‐scores (*p* = 0.028 and *p* = 0.005, respectively). These findings support the importance of first trimester iodine status and suggest need for preconception supplementation beyond salt iodization alone.

**Key messages**

- Maternal iodine status was better at the end of the first trimester, and prevalence of I/Cr < 150 μg/g was lower in women who had started nutrition supplementation at least 3 months prior to conception compared with unsupplemented women.
- Maternal iodine status at the end of the first trimester, but not in the third trimester, was positively associated with birth length and head circumference.
- In two of three study sites, more than a third of the women who received supplementation prior to conception had urinary I/Cr ≥ 250 μg/g but with no evidence of adverse effects.

## 1 INTRODUCTION

Iodine (I) is an essential micronutrient that is required for the synthesis of the thyroid hormones triiodothyronine and thyroxin. Iodine deficiency remains a major public health issue for pregnant women and young children throughout the world. The greatest cause of preventable brain damage in childhood is iodine deficiency (Rohner et al., 2014). Despite worldwide efforts to eliminate iodine deficiency, particularly through fortification programmes, recent estimates indicate approximately 26% of the world's households do not have sufficient iodine intake (UNICEF, 2015; World Health Organization, 2004). Iodine deficiency during pregnancy can have devastating effects on foetal and neonatal development, including resultant thyroid failure leading to irreversible abnormalities in brain development and function.

Maternal iodine status is particularly important during the first trimester of pregnancy because the embryo relies solely on maternal iodine and thyroid function for normal development. Thyroid hormone deficiency in the first trimester of gestation has been found to have detrimental effects on neuronal migration, cortical lamination and callosal projections in rodent development and on visual attention and visual processing in humans (Zoeller & Rovet, 2004). The recommended daily allowance of iodine during pregnancy ranges from 250 to 500 μg/day (Institute of Medicine & Food and Nutrition Board, 2001; WHO Secretariat et al., 2007; World Health Organization, 2007). Typically, micronutrient supplementation in pregnancy is initiated after the first trimester and continues throughout pregnancy, but this bypasses the critical period of development early in gestation. Studies of iodine supplementation during pregnancy and effects on early childhood neurodevelopment have reported inconsistent findings (Bell et al., 2016; Bleichrodt & Born, 1994; Delange, 1985, 2001; Dineva et al., 2020; Gowachirapant et al., 2017; Melse‐Boonstra & Jaiswal, 2010). Furthermore, few studies have examined the role of preconception iodine supplementation on birth outcomes and child neurodevelopment (Robinson et al., 2018).

The principal objective of this study, an a priori secondary analysis of the primary ‘Women First’ (WF) Preconception Maternal Nutrition trial (Hambidge et al., 2014), was to assess the impact of iodine supplementation started in the preconception period on maternal iodine status. A second goal was to determine if iodine status of this population was associated with newborn anthropometric outcomes. The primary WF trial demonstrated significant improvements in birth length and weight for the newborns of women who received the nutrition intervention, small‐quantity lipid‐based nutrient supplement (SQ‐LNS), starting either before conception or starting at the end of the first trimester of pregnancy compared with women who received no study intervention (Hambidge et al., 2019). For the current analysis, we hypothesized that starting iodine supplementation provided in the SQ‐LNS during the preconception period would result in significantly higher urinary iodine to creatinine ratios (I/Cr) at 12 and 34 weeks gestation, representing improved maternal iodine status, which would be positively correlated with newborn anthropometry.

## 2 METHODS

### 2.1 Study design

Urinary I/Cr (μg/g) for women in low resource settings in rural Guatemala, Pakistan and India were measured during the first and third trimesters, and the potential association of iodine status with birth anthropometric outcomes was determined. The daily iodine supplement was provided in an SQ‐LNS.

The WF trial was an individually randomized, nonmasked, multisite controlled trial conducted in rural or semirural locations in four low resource settings in Guatemala, India, Pakistan and Democratic Republic of Congo (DRC) (Hambidge et al., 2014). Women were identified through household surveys, local health centres and local advertising. Community sensitization meetings to explain the study to prospective participants were held prior to study initiation. Due to limitations of cold chain capacity in the DRC, data presented here represent only the first three of these participating sites. The primary outcome of the trial was to evaluate the impact of maternal SQ‐LNS initiated prior to conception on foetal growth (Hambidge et al., 2019). At the time of enrolment (baseline), non‐pregnant women were randomized to one of three intervention arms: Arm 1 commenced the supplement immediately after randomization and continued for ≥ 3 months prior to conception until delivery, Arm 2 commenced the same intervention late in the first trimester and continued until delivery and Arm 3 received no study supplements. All women were followed via biweekly visits from enrolment through 6 months post‐partum. Of 5646 women who were consented and randomized, 2442 women became pregnant and had the primary outcome for newborns, including 802, 835 and 805 for Arms 1, 2 and 3, respectively (Figure S1).

The SQ‐LNS provided 250‐μg iodine daily. Longitudinal single spot urine and serum samples were obtained at 12 and 34 weeks gestation from approximately 100 women per arm per site. These numbers were considered to be adequate for the goals of the study and the maximum that could be collected, stored, transported and analysed within the resources of the individual sites and the WF trial. Participants in Arms 1 and 2 provided samples at both time points whereas participants in Arm 3 only provided samples at 34 weeks. For Arm 2, the first urine sample was collected prior to initiation of the daily study supplement; this group thus served as control group at this time point. The India site was only able to collect samples from women in Arms 1 and 2.

### 2.2 Subjects

For all arms, each participant who entered the pregnancy phase of the study was consented to provide urine collections, blood and other biospecimens (unrelated to this report). Hence, the women in the current study were primarily those who were the first to conceive. For the analysis of iodine status in relation to birth measurements, only those newborns with gestational age determined by first trimester ultrasounds were used. This represented approximately two thirds of the mother–infant dyads, with equal distribution among the three arms (Figure S1). Details of procedures for newborn anthropometry (length, weight and head circumference), all obtained within 48 h of delivery, have been reported previously (Hambidge et al., 2019). For newborns with first trimester ultrasound‐determined gestational age, measurements were converted to gestational age adjusted length‐, weight‐, head circumference‐ and weight to length ratio‐for‐age *z*‐scores (LAZ, WAZ, HCAZ and WLRAZ, respectively) using INTERGROWTH‐21st foetal growth charts (Papageorghiou et al., 2014). Data were collected between December 2013 and December 2016.

### 2.3 Sample collection and laboratory procedures

Spot urine samples (~30 ml) for iodine and creatinine were collected at 12 (Arms 1 and 2) and 34 weeks gestation (Arms 1, 2 and 3, excluding Arm 3 in India). Twenty‐four‐hour urine collections were not feasible due to the remoteness of many of the participants' homes, the common practice of women working in fields and the absence of centralized clinical facilities where overnight collections could be supervised. Specimens were collected in containers locally sourced by each site with ~5‐ml aliquot transferred to a cryovial. Maternal blood samples for thyroid‐stimulating hormone (TSH) were collected at the same time points and serum transferred to cryovials. Samples were stored in −80°C freezers located at each site until shipment on dry ice to the University of Colorado Pediatric Nutrition Laboratory for analyses. Upon receipt, all samples were stored at −80°C until analysed.

Urinary iodine concentration was measured by inductively coupled plasma mass spectrometry (Agilent Technologies 7700, Santa Clara, CA) following the CDC Urinary Iodine Method Protocol (CDC Environmental Health, 2007) with modifications. The addition of base urine to standards, controls and samples was replaced with diluent, and the water volume in blank was reduced to 100 μl. Validation studies showed that these modifications did not affect accuracy or precision of the method (unpublished data). Calibration standards were prepared with Supelco TraceCert™ ICP Iodide standard solution and internal standard solution with TraceCert™ ICP Tellurium standard solution (#41271 and #78358, respectively, Sigma‐Aldrich, St. Louis, MO). Internal controls, including an in‐house urine pool (low value), spot urine (high value) and SeroNorm™ Trace Elements Urine Levels 1 and 2 RUO (#210613 and 210713, respectively, Accurate Chemical and Scientific Corporation, Westbury, NY) were randomized and inserted after each 10 samples within every analytical run. The lower limit of quantitation of the method was 10 μg/L with seven results obtained below this limit. These results were reported as 9 μg/L for statistical analyses. Within assay precision was 4.6%; between assay precision ranged between 4% and 5% for the internal controls. We participate in the CDC EQUIP standardization programme for urinary iodine testing, which yielded scores of 100% on the performance summary (three rounds between May 2019 and February 2020). To minimize potential batch effects, samples from Guatemala and Pakistan were balanced across runs by randomizing within site, arm and longitudinal time point using Excel. Samples from India were not randomized but instead were run chronologically by participant ID, starting with 12‐ and then 34‐week time points; the study design, however, randomized participant ID across arms, which also minimized batch effects.

Urinary creatinine concentrations were measured in the same batch order as iodine using a colorimetric assay (Creatinine Urinary Colorimetric Assay Kit 500701, Cayman Chemical Company, Ann Arbor, MI) per manufacturer's protocol. Two controls, a urine pool and the Acusera Assay Urine Quality Control Level 2 (Item No AU2352, Randox Laboratories, Crumlin, UK), were analysed on every plate to monitor assay performance. The lower limit of quantitation was 1.8 mg/dl; within day precision was 4%, and between day precision was 6.3% and 7.9% for the Acusera control and urine pool, respectively.

TSH was measured in maternal serum from Guatemala and Pakistan using a one‐step sandwich method (Beckman Coulter, Brea, CA) by the University of Colorado Hospital CTRC Laboratory (Aurora, CO). The sensitivity of the method is 0.01 μIU/ml; within day and between day precisions were both < 2%. TSH in the maternal serum samples from India were measured by the KLE Laboratory via chemiluminescence immunoassay (Siemens Advia XP) with between run and within run variability < 9%.

Chi‐squared tests of independence were used to detect whether the number of women in each arm differed by batch for both iodine and creatinine assays. Pirate plots by batch were used to visualize iodine and creatinine.

### 2.4 Data analysis and statistical methods

Women were assigned to categories of iodine status based on the following I/Cr: I/Cr < 50, 50–149, 150–249, ≥ 250–499 and ≥ 500 μg/g (Torlinska et al., 2018). Urinary iodine concentration (μg/L) was also determined and similarly categorized. For most analyses, an all‐site regression model adjusting for site and regressions stratified by site were performed. Stratified regressions by site were not performed for dichotomous newborn anthropometry measures because the models were unstable due to low cell counts. All analyses adjusted for urinary iodine values ≤ 25 μg/g as these values were found to be less reliable. Separate analyses were performed for I/Cr at 12 and 34 weeks.

Statistical Software R v3.6.2 was used for all statistical analyses (R Core Team & R Foundation for Statistical Computing, 2018).

#### 2.4.1 Association between I/Cr or TSH and arm

Multiple linear regression was used to investigate the relationship between continuous I/Cr or TSH as the outcome and arm as the primary predictor using the *glm* function in base R. Given right skewness in the distributions of I/Cr and TSH, log10 transformation was used for I/Cr and TSH. Results were untransformed to interpret the results. Multinomial logistic regression was used to investigate the relationship between I/Cr category as the outcome and arm as the primary predictor using the *multinom* function from the nnet package (v7.3‐12) (Venables & Ripley, 2002).

#### 2.4.2 Association between TSH and I/Cr

Multiple linear regression was used to investigate the relationship between continuous TSH as the outcome and I/Cr category as the primary predictor using the *glm* function in base R. The models were adjusted for arm and for I/Cr ≤ 25.

#### 2.4.3 Association between I/Cr and birth outcomes

To investigate the relationship between continuous birth outcomes and maternal iodine status, a multiple linear regression model was used with continuous newborn anthropometry measures after adjusting for gestational age (i.e., LAZ, WAZ, HCAZ and WLRAZ) as the outcome and either I/Cr continuous or I/Cr categories as the primary predictor using the *glm* function in base R. A log‐binomial regression was used to investigate the relationship between dichotomous birth outcome and I/Cr, with dichotomous gestational age adjusted newborn anthropometry measures (i.e., LAZ < −2, WAZ < −1.287 [consistent with cut‐off for small‐for‐gestational age], HCAZ < −2 or WLRAZ < −2) as the outcome of the regression and either I/Cr continuous or I/Cr categories as the primary predictor using the *glm* function in base R. All models included arm as a covariate.

#### 2.4.4 Outliers and model robustness

Observations three interquartile units above or below the third and first quartiles, respectively, were defined as outliers. Outliers were identified separately for iodine, creatinine and I/Cr values. All outliers, all of which were high extremes, were removed from the primary analyses. The outliers comprised 2.9% of the total number of samples.

To assess stability of results, secondary analyses were performed adjusting for cluster or including outliers. Analyses with and without outliers did not differ. Secondary analyses including cluster were not performed for dichotomous newborn anthropometry measures and I/Cr categories due to the model being unstable when cluster was included.

#### 2.4.5 Multiple testing

Nominal statistical significance was assessed at *p* value of 0.05. Statistical significance adjusting for multiple testing within 12 weeks and within 34 weeks models was assessed using the Bonferroni correction at 0.05/18 = 0.0028 where the 18 primary models include association of eight birth outcomes (LAZ < −2, WAZ < −1.287, HCAZ < −2 and WLRAZ < −2) and arm by both continuous I/Cr and categorical I/Cr. Post hoc analyses were performed using the *emmeans* function from the emmeans R package v1.4.4. Tukey adjustment for multiple testing was used for post hoc comparison of categorical variables (i.e., Arms 1, 2 and 3 at 34 weeks and categorical I/Cr groups).

#### 2.4.6 Mediation analyses

For continuous newborn anthropometry measures with a nominal statistical significance (*p* < 0.05) with continuous I/Cr for the all‐site model at either 12 or 34 weeks, we proceeded with mediation analysis to determine whether I/Cr is a potential mediator of the relationship between arm and newborn anthropometry measures. Mediation analysis was performed using the *mediate* function from the mediation R package v4.5.0 (Tingley et al., 2014) setting robustSE to TRUE and using 1000 simulation replicates. As with the other primary analyses, outliers were excluded.

### 2.5 Ethical considerations

The project was approved by the Colorado Multiple Institutional Review Board, University of Colorado, the local and/or national ethics committees for each of the three sites (registered with US Office of Human Research Protection and with Federalwide Assurance in place) and the data coordinating centre. Written informed consent was obtained from all participants prior to participation in the study. Throughout the intervention phase of the trial, a data monitoring committee designated by the *Eunice Kennedy Shriver* National Institute of Child Health and Human Development monitored safety of the trial. The study protocol is available online: https://www.ncbi.nlm.nih.gov/pmc/articles/PMC4000057/, and the trial is registered as ClinicalTrials.gov #NCT01883193 at https://clinicaltrials.gov/ct2/show/NCT01883193?term=01883193%24rank=1.

## 3 RESULTS

Baseline description of women who provided the urine samples for these analyses is in Table 1 and Figure S1 according to arm, each site and stage of gestation.

**TABLE 1:** Maternal baseline characteristics and infant outcomes by arm and time point, by combined and individual site

	12 weeks gestation		34 weeks gestation		
Variable	Preconception supplement (Arm 1)^a^	Preinitiation of supplement (Arm 2)^a^	Preconception supplement (Arm 1)	Prenatal supplement (Arm 2)	Control (Arm 3)^a^
Women with I/Cr, *n*					
Combined sites	322	319	305	306	231
Guatemala	109	112	108	111	114
India^b^	110	104	90	83	—
Pakistan	103	103	107	112	117
Maternal age, *n* (%)					
Mean ± SD	23.3 ± 6.3	23.3 ± 3.9	23.3 ± 6.3	23.7 ± 3.9	24.1 ± 4.3
<20 years	63 (20)	54 (17)	59 (19)	47 (15)	38 (17)
≥20 years	259 (80)	265 (83)	246 (81)	259 (85)	192 (83)
BMI (kg/m^2^)					
Mean ± SD	21.7 ± 2.9	21.8 ± 4.4	21.8 ± 2.9	21.7 ± 4.4	22.1 ± 4.5
<18.5	87 (27)	75 (24)	82 (27)	77 (25)	46 (20)
18.5–24.9	166 (52)	176 (55)	157 (51)	163 (53)	132 (57)
≥25	69 (21)	68 (21)	66 (22)	66 (22)	52 (23)
Maternal education, *n* (%)					
No formal schooling	101 (31)	94 (29)	101 (33)	100 (33)	110 (48)
Primary	88 (27)	106 (33)	88 (29)	100 (33)	93 (40)
Secondary	133 (41)	119 (37)	116 (38)	106 (35)	27 (12)
Parity, *n* (%)					
0 (nulliparous)	88 (27)	63 (20)	81 (27)	57 (19)	38 (17)
1	101 (31)	110 (34)	98 (32)	103 (34)	65 (28)
≥2	133 (41)	146 (46)	126 (41)	146 (48)	127 (55)
SES,^c^ *n* (%)					
None (0 present)	3 (1)	1 (0)	4 (1)	1 (0)	5 (2)
1–2 present	72 (22)	69 (22)	74 (24)	67 (22)	57 (25)
3–4 present	168 (52)	164 (51)	154 (50)	163 (53)	119 (52)
5–6 present	79 (25)	85 (27)	73 (24)	75 (25)	49 (21)
Infant birth outcomes,^d^ mean ± SD					
*n*	262	260	260	259	177
Length (cm)	47.6 ± 2.1	47.8 ± 2.3	47.6 ± 2.2	47.8 ± 2.2	47.2 ± 2.4
LAZ	−0.71 ± 0.97	−0.60 ± 1.01	−0.74 ± 0.99	−0.63 ± 1.00	−0.89 ± 1.10
Weight (g)	2798 ± 398	2818 ± 453	2790 ± 40	2828 ± 437	2787 ± 416
WAZ	−0.91 ± 0.88	−0.87 ± 0.95	−0.93 ± 0.89	−0.87 ± 0.95	−0.89 ± 0.89
Head circumference (cm)	33.1 ± 1.3	33.1 ± 1.5	33.1 ± 1.3	33.1 ± 1.4	33.0 ± 1.6
HCAZ	−0.43 ± 1.00	−0.43 ± 1.10	−0.46 ± 1.00	−0.45 ± 1.10	−0.49 ± 1.10
WLRAZ	−1.26 ± 1.27	−1.19 ± 1.35	−1.26 ± 1.26	−1.20 ± 1.35	−1.11 ± 1.20

Abbreviations: BMI, body mass index; HCAZ, head circumference‐for‐age *z*‐score; I/Cr, iodine to creatinine ratio; LAZ, length‐for‐age *z*‐score; SES, socio‐economic status; WAZ, weight‐for‐age *z*‐score.

^a^ Arm 1 commenced the supplement ≥ 3 months prior to conception and continued through delivery; Arm 2 commenced the same intervention late in the first trimester (after sample collection) and continued until delivery; Arm 3 (Control) received no study supplements.

^b^ Control arm (Arm 3) is not available for India at 34 weeks.

^c^ The SES tally provides the number of indicators available from the following list: electricity, improved water source, sanitation (flush toilet), man‐made flooring, improved cooking fuels and household assets.

^d^ Participants for whom gestational age was determined by first trimester ultrasound.

For comparison purposes to other data sets, the medians of urine iodine concentration (UIC, μg/L) by arm are presented in the supplemental data (Figure S2a,b). At 12 weeks gestation, adjusted mean I/Cr for combined sites was significantly higher for Arm 1 versus Arm 2 (Table 2). Similarly, a statistically significant higher adjusted mean I/Cr for Arm 1 versus Arm 2 was observed for both Guatemala and India but not for Pakistan.

**TABLE 2:** Adjusted iodine to creatinine ratio (I/Cr, μg/g) and thyroid‐stimulating hormone (TSH, IU/L) by arm at 12 weeks gestation for combined and individual sites

Site	Arm^a^	*N*	Adj mean (95% CI)	Fold change Arm 1/Arm 2 (95% CI)	*p* value^b^
I/Cr (μg/g)					
Combined sites	1	322	203 (189, 217)	1.24 (1.13, 1.37)	<0.0001
	2	319	163 (152, 175)		
Guatemala	1	109	224 (197, 254)	1.39 (1.16, 1.66)	<0.001
	2	112	161 (142, 183)		
India	1	110	222 (196, 252)	1.44 (1.20, 1.72)	<0.0001
	2	104	155 (136, 177)		
Pakistan	1	103	162 (147, 180)	0.92 (0.81, 1.06)	0.25
	2	103	176 (159, 194)		
TSH (IU/L)					
Combined sites	1	322	1.08	1.04 (0.90, 1.19)	0.620
	2	319	1.05		
Guatemala	1	109	1.32	0.99 (0.83, 1.19)	0.120
	2	112	1.33		
India	1	110	1.00	1.07 (0.82, 1.40)	0.140
	2	104	0.93		
Pakistan	1	103	0.96	1.01 (0.80, 1.28)	0.909
	2	103	0.94		

^a^ Arm 1 commenced the supplement ≥ 3 months prior to conception and continued through delivery; Arm 2 commenced the same intervention late in the first trimester (after sample collection) and continued until delivery.

^b^ Multiple linear regression was used with continuous I/Cr or TSH (log10 transformed) as the outcome and arm as the primary predictor and removing outliers. I/Cr models also adjusted for iodine ≤ 25 μg/L. All results were untransformed to enable interpretability in standard units. Site was adjusted for in the combined site analysis.

**FIGURE 1:**
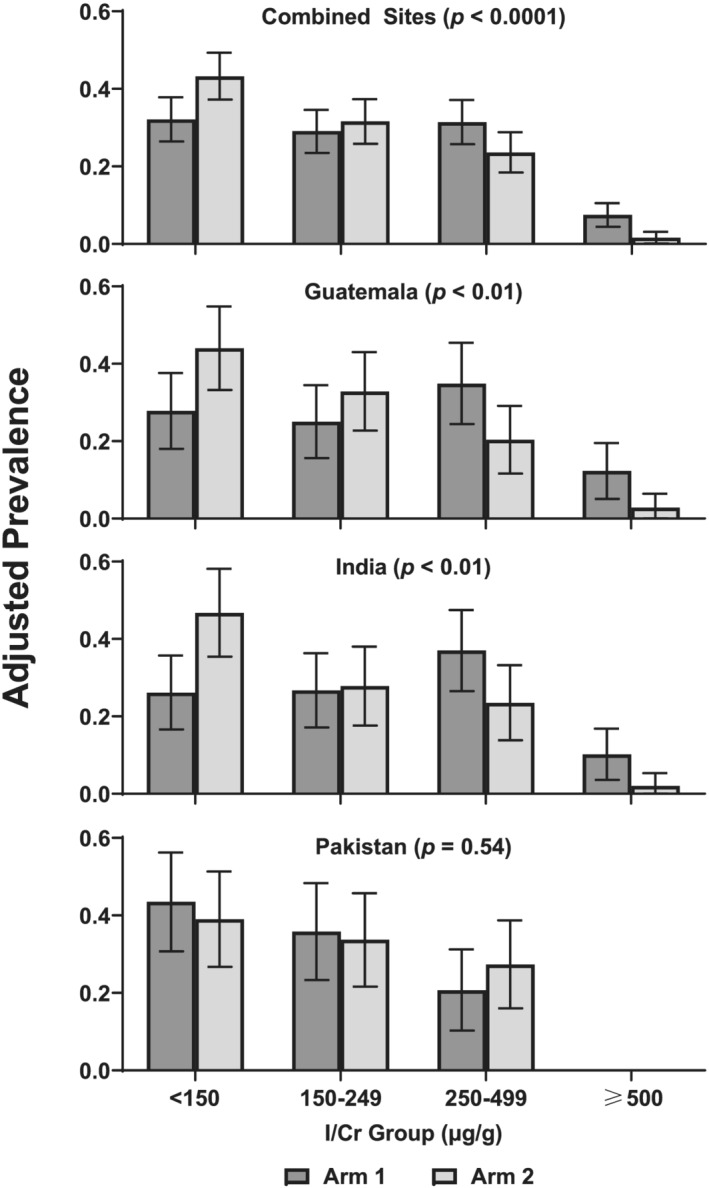
Iodine to creatinine ratio (I/Cr, μg/g) by categories of maternal status at 12 weeks gestation by site and by arm. Multinomial logistic regression was used to investigate the relationship between I/Cr category as the outcome and arm. All analyses were adjusted for iodine ≤ 25 μg/L. The combined site analysis was also adjusted for site. Outliers were removed prior to analysis. Data are presented as mean (95% CI). Arm 1 commenced the supplement ≥3 months prior to conception, and Arm 2 commenced the same intervention late in the first trimester (after sample collection); both arms discontinued supplement at delivery

Because of the small number of women who had results in the < 50 μg/g category, the lower two categories were combined for subsequent categorical analyses. The distributions of I/Cr categories at 12 weeks differed by arm overall (*p <* 0.0001) and for Guatemala (*p* < 0.01) and India (*p* < 0.01) but not Pakistan (*p* = 0.54) (Figure 1). The adjusted prevalence (95% CI) of I/Cr < 150 was lower for Arm 1 versus Arm 2 in India (26% [95% CI: 17%, 30%] vs. 47% [35%, 58%]) and Guatemala (30% [18%, 38%] vs. 44% [33%, 55%]). The adjusted prevalence of I/Cr < 150 in Pakistan was generally higher than India and Guatemala but similar by arm (44% [31%, 56%] vs. 39% [27%, 51%] in Arms 1 and 2, respectively). Adjusted prevalence of I/Cr ≥ 500 also differed by arm and by site; no women had I/Cr ≥ 500 in Pakistan once outliers were removed: 12% (5%, 20%) for Arm 1 and 3% (0%, 6%) for Arm 2 in Guatemala and 10% (4%, 17%) in Arm 1 and 2% (0%, 5%) in Arm 2 for India (Figure 1).

The adjusted means by arm and by site at 34 weeks are shown in Table 3. A statistically significant difference in the adjusted mean I/Cr for both intervention arms compared with the control arm was observed for Guatemala but not for Pakistan; no data for Arm 3 in India were available for comparison with the intervention arms. No statistical differences were observed between Arm 1 versus Arm 2 for any of the three sites at 34 weeks (Figure S3).

**TABLE 3:** Adjusted iodine to creatinine ratio (I/Cr, μg/g) and thyroid‐stimulating hormone (TSH, IU/L) by arm at 34 weeks gestation for combined and individual sites

				Fold change			
Site	Arm^a^	*N*	Adj mean I/Cr (95% CI)	Arm comparison	Ratio (95% CI)	Pairwise *p* value^b^	Global *p* value^b^
I/Cr (μg/g)							
Combined sites	1	305	235 (220, 252)	1/2	0.93 (0.84, 1.02)	0.24	<0.0001
	2	306	254 (238, 272)	1/3	1.17 (1.06, 1.31)	<0.01	
	3	231	200 (184, 218)	2/3	1.27 (1.14, 1.41)	<0.0001	
Guatemala	1	108	268 (239, 301)	1/2	1.00 (0.85, 1.18)	1.00	<0.0001
	2	111	267 (239, 299)	1/3	1.47 (1.25, 1.72)	<0.0001	
	3	114	182 (163, 204)	2/3	1.47 (1.25, 1.72)	<0.0001	
India	1	90	252 (219, 289)	1/2	0.90 (0.73, 1.10)	0.29	0.29
	2	83	281 (242, 325)				
Pakistan	1	107	193 (175, 213)	1/2	0.87 (0.76, 1.00)	0.12	0.14
	2	112	221 (201, 243)	1/3	0.95 (0.83, 1.08)	0.68	
	3	117	204 (186, 224)	2/3	1.08 (0.95, 1.23)	0.46	
TSH (IU/L)							
Combined sites	1	305	1.90 (1.79, 2.01)	1/2	0.98 (0.91, 1.07)	0.90	0.29
	2	306	1.93 (1.82, 2.05)	1/3	0.93 (0.84, 1.02)	0.27	
	3	231	2.05 (1.89, 2.21)	2/3	0.95 (0.86, 1.04)	0.47	
Guatemala	1	108	2.05 (1.87, 2.24)	1/2	1.03 (0.91,1.17)	0.88	0.53
	2	111	1.98 (1.81, 2.17)	1/3	0.96 (0.84, 1.09)	0.78	
	3	114	2.14 (1.95, 2.35)	2/3	0.93 (0.81, 1.06)	0.49	
India	1	90	1.93 (1.73, 2.15)	1/2	0.91 (0.78, 1.06)	0.22	0.22
	2	83	2.13 (1.90, 2.39)				
Pakistan	1	107	1.73 (1.56, 1.92)	1/2	1.01 (0.88, 1.17)	0.98	0.54
	2	112	1.71 (1.54, 1.89)	1/3	0.94 (0.82, 1.08)	0.65	
	3	117	1.84 (1.67, 2.03)	2/3	0.93 (0.81, 1.07)	0.54	

^a^ Arm 1 commenced the supplement ≥ 3 months prior to conception and continued through delivery; Arm 2 commenced the same intervention late in the first trimester (after sample collection) and continued until delivery; Arm 3 (Control) received no study supplements. No Arm 3 data were available in the Indian site.

^b^ Multiple linear regression was used with continuous I/Cr or TSH (log10 transformed) as the outcome and arm as the primary predictor and removing outliers. I/Cr models also adjusted for iodine ≤ 25 μg/L. All results were untransformed to enable interpretability in standard units. Site was adjusted for in the combined site analysis.

**FIGURE 2:**
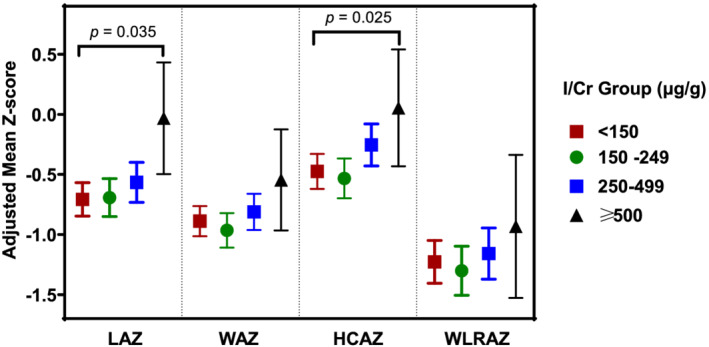
Categorical iodine to creatinine ratio (I/Cr, μg/g) groups and continuous birth outcomes for combined sites at 12 weeks gestation. Multiple linear regression models were used with gestational adjusted continuous newborn anthropometry measures as the outcome and I/Cr categories as the primary predictor and adjusting for arm. Outliers were removed prior to analyses, and low iodine values were adjusted for by including an indicator for iodine ≤ 25 μg/L as a predictor in the linear model. Data are presented as adjusted mean (95% CI). *N*_<150_ = 215–216; *N*_150–249_ = 148–150; *N*_250–499_ = 136–137; *N*_≥500_ = 18. Abbreviations: HCAZ, head circumference‐for‐age *z*‐score; LAZ, length‐for‐age *z*‐score; LWRAZ, length to weight ratio‐for‐age *z*‐score; WAZ, weight‐for‐age *z*‐score

For the all‐site analysis at 12 weeks, adjusting for site and arm, there was a small, nominally significant association between I/Cr and birth LAZ (*p* = 0.028) and between I/Cr and birth HCAZ (*p* < 0.01) but not for other continuous or dichotomous newborn anthropometric measures (Figures 2 and S4) The adjusted effect size for LAZ was +0.07 per 100 unit increase in I/Cr and was primarily driven by Guatemalan data. The adjusted effect size for HCAZ was +0.09 per 100 unit increase in I/Cr and was driven by India (0.18 per 100 I/Cr, *p* < 0.01) and somewhat by Guatemala (0.10 per 100 I/Cr, *p* = 0.034) but was not observed in Pakistan. No statistically significant (i.e., *p* < 0.05) associations were observed between the 34‐week I/Cr and newborn anthropometric outcomes for the all sites analysis (Figure S5).

Mediation analyses revealed that I/Cr at 12 weeks for combined sites was a nominally significant mediator for LAZ (*p* = 0.046) and HCAZ (*p* = 0.016). No individual sites showed strong or statistically significant evidence of mediation for LAZ. The HCAZ mediation result is driven primarily by India (*p* = 0.023) and partially by Guatemala (*p* = 0.06) (Figures S6a,b).

TSH was significantly associated with I/Cr category at 12 weeks for India (*p* < 0.01) but not for Guatemala (*p* = 0.92) or Pakistan (*p* = 0.42). For India, adjusted TSH level increased as I/Cr increased. TSH did not differ significantly by arm at 12 weeks or by arm or I/Cr group at 34 weeks for any of the sites.

## 4 DISCUSSION

Iodine is an outstanding example of an individual nutrient for which adequate maternal status is critical from the earliest days of embryonic development because maternal thyroid hormones are vital for the formation and growth of nerve cells starting as early as the second month of gestation (Dineva et al., 2020; Harding et al., 2017; Prado & Dewey, 2014). In this study, the mean maternal urine iodine, a measure of both recent iodine intake and of iodine status, was significantly higher at the end of the first trimester for women in Arm 1, who started the study daily nutrition supplement at least 3 months prior to conception, compared with those in Arm 2, who had not yet started it at the time of iodine measurement. The preconception arm also had a significantly lower prevalence of mothers in the low (I/Cr < 150) category at 12 weeks gestation in two of the three sites. In the third trimester, iodine status for both Arms 1 and 2, the latter having been on the supplement for approximately 20 weeks, were higher than for the women in the control Arm 3, who received no study supplements. We saw nominally significant positive associations between I/Cr and LAZ and HCAZ, driven primarily by Guatemala and India at 12 weeks, and no significant association between iodine status at 34 weeks and birth outcomes.

The primary purpose of the WF trial was to determine the value of improving maternal nutrition prior to conception. We have identified only two previous reports of iodine supplementation prior to conception, and these were single intramuscular injections with no urine iodine data (Kevany et al., 1969; Pharoah et al., 1971). The finding of a substantially reduced prevalence of iodine deficiency in the current study is of special note and illustrates the benefits of improving maternal iodine status starting prior to conception. The value of this finding is supported by results from a large prospective cohort of mothers and offspring that indicated a positive association between preconception maternal iodine status and cognitive function in the offspring at 6–7 years of age (Robinson et al., 2018). In contrast, meta‐analyses of iodine supplementation initiated during pregnancy found no effect on child neurodevelopment, possibly partially attributable to the timing the interventions (Dineva et al., 2020; Levie et al., 2019).

Whereas the urine iodine data were similar for Guatemala and India, those for Pakistan were quite different and tended toward more values in lowest range. Of note was the failure of the iodine supplement commenced prior to conception to reduce the prevalence of I/Cr in the < 150 category, which remained close to 50%. Ninety per cent of ingested iodine is promptly excreted in the urine if iodine status is normal or near normal. However, excretion will be substantially reduced with chronic iodine deficiency. The Pakistan data are consistent with a degree of chronic iodine insufficiency, though it is somewhat unexpected that a pre‐existing deficiency would not have been corrected after at least 6 months of supplementation. Similar observations, however, were reported for an LNS supplementation trial during pregnancy in Bangladeshi women who had very low iodine urine concentrations in the second trimester that remained low after approximately 4 months of supplementation (Mridha et al., 2017). Another pregnancy LNS intervention in a population with higher but marginal baseline (second trimester) UICs found significant (though modest) improvements by 36 weeks gestation (Adu‐Afarwuah et al., 2018), consistent with our findings for Arm 2 compared with the control Arm 3.

By country surveillance data, Pakistan historically has had somewhat lower rates of iodine consumption by household (60–80%) compared with Guatemala or India (> 80% for both) (UNICEF, 2017). Local surveillance data were not available for our regions or communities. In addition, the population in Pakistan has been reported to have a lower mean urinary iodine concentration compared with Guatemala or India (Iodine Global Network, 2017, 2019). TSH concentrations were normal in all sites, including Pakistan. While TSH is an insensitive biomarker of iodine status, it is a sensitive biomarker of thyroid function, indicating that iodine deficiency in the Pakistan cohort was not sufficient to result in overt hypothyroidism.

In Guatemala and India, slightly over a third of the participants had I/Cr considered more than adequate or excessive, and in both settings, the preconception arm had a higher percentage in these two categories. The amount of iodine in the supplement met recommendations for pregnancy by the WHO Secretariat et al. (2007), but in settings with relatively effective fortification programmes, this amount likely resulted in a rightward shift in the distribution. In Pakistan, where the levels were generally lower, only approximately 25% of the levels were in the two high categories, with no significant difference by arm. One report with a large sample size found excessive iodine intakes, reflected by UICs over 500 μg/L, in early pregnancy in an iodine‐sufficient population, were associated with significantly increased risk of hypothyroidism (Shi et al., 2015). The TSH data in our study did not support an adverse impact on thyroid function, but our relatively small sample size may have limited capacity to detect such an effect. In contrast, however, I/Cr levels in the highest category at 12 weeks were associated with statistically greater birth length and head circumference, and mediation analysis supported possible causality. This finding warrants replication and raises an important perspective to be considered in assessment of optimal maternal iodine intake and status, especially in highly vulnerable populations.

The primary limitation to this secondary analysis was the availability of a single spot urine at each of the two times of gestation. Although inadequate for assessment of the iodine intake or status of any one individual, the single spot urine specimen has been accepted for assessment of populations or smaller groups as in this study (Harding et al., 2017; Robinson et al., 2018; Rohner et al., 2014; Zimmermann & Andersson, 2012). To reduce the variation in effects of dilution in spot urine samples and to provide a better assessment of individuals' iodine status, we utilized I/Cr as a marker for maternal iodine status. The main limitations of this approach are that creatinine concentrations may vary by diet and time of day (Soldin, 2002) and that compromised general nutrition status is associated with lower creatinine excretion due to overall lower muscle mass. This may have been relevant in some of our participants, including especially in India and Pakistan where over a third of the women were underweight (BMI < 18.5 kg/m^2^) at baseline (Hambidge et al., 2017). This could have falsely raised the I/Cr and thus underestimated the prevalence of low iodine status in our cohort. The subsample of participants who provided the urine samples was not randomly selected, which could limit reliability and generalizability of our findings. However, no differences were identified between the women who contributed biological specimens and those who entered the pregnancy phase of the trial after the target numbers of biological sample were achieved. Additionally, the randomized trial design supports our primary emphasis on comparisons among arms. Despite these limitations in the urine collections and the selection of the study population, the results support benefits of improving women's nutritional status several months prior to conception.

Lastly, with maternal iodine status and thyroid function being crucial role in embryogenesis (Zoeller & Rovet, 2004), it is important that maternal iodine status be sufficient very early in the first trimester of gestation. Future investigations are merited to determine the effects of maternal iodine and thyroid status, specifically in the period prior to conception, on neurodevelopment in the offspring of the participants in this study.

## 5 CONCLUSION

To the best of our knowledge, this randomized controlled trial is the first to report the effects of maternal daily iodine supplementation initiated prior to conception in low resource settings with populations with marginal iodine intake and status despite national salt fortification programmes. Maternal iodine status at the end of the first trimester was improved with supplementation started in preconception period, and iodine status was nominally associated with greater birth length and head circumference. Investigations on growth and neurodevelopmental outcomes in the children of mothers in the lowest iodine versus higher groupings will be important to assess the persistence of these early gestational effects.

Although enormous progress has been achieved with universal salt iodization programmes, these findings suggest insufficient local awareness of the suboptimal iodine status of women of child‐bearing age within countries that have existing national iodization guidelines. Additionally, the results suggest a need for improvement of iodine status of women of reproductive age beyond salt fortification alone in these settings. The absence of any significant associations of third trimester iodine status with birth anthropometry adds to the accumulating evidence that nutritional supplementation initiated several months prior to conception has more beneficial effects compared with supplementation initiated after the first trimester.

## CONFLICTS OF INTEREST

The authors declared no conflicts of interest.

## CONTRIBUTIONS

AEY, JFK, CU, JLW, SAA, SS, AG, LF, MSS and SSG performed the research. NFK and KMH designed the research study. AEH analysed the data. AEY, NFK, KMH and AEH wrote the paper. All authors have read and approved the final manuscript.

## Supporting information

**Figure S1** Consort diagram of Women First participants in Guatemala, India, and Pakistan

**Figure S2** Urinary Iodine Concentration (UIC) vs Iodine:Creatinine Ratio (I/Cr) at 12‐ and 34‐wk gestation by Arm

**Figure S3** Iodine to creatinine ratio (I/Cr, μg/g) by categories of maternal status at 34 weeks gestation by site and by arm

**Figure S4** Categorical iodine to creatinine ratio (I/Cr, μg/g) groups and dichotomous birth outcomes for combined sites at 12 weeks gestation

**Figure S5** Categorical iodine to creatinine ratio (I/Cr, μg/g) groups and continuous birth outcomes at 34 weeks for combined sites

**Figure S6** a and b Mediation analyses at 12 weeks

## Data Availability

Data described in the manuscript are available from the corresponding author upon reasonable request.
