# Supplementary material for: Improved first trimester maternal iodine status with preconception supplementation: The Women First Trial
Source: Matern Child Nutr. 2021 May 25;17(4):e13204. doi: 10.1111/mcn.13204 (PMC8476419; doi:10.1111/mcn.13204)
Supplement: Supplementary file 1 — Figure S1 Consort diagram of Women First participants in Guatemala, India, and Pakistan [file MCN-17-e13204-s004.pdf]

Supplemental Figure 1. Consort diagram of Women First participants in Guatemala, India, and Pakistan

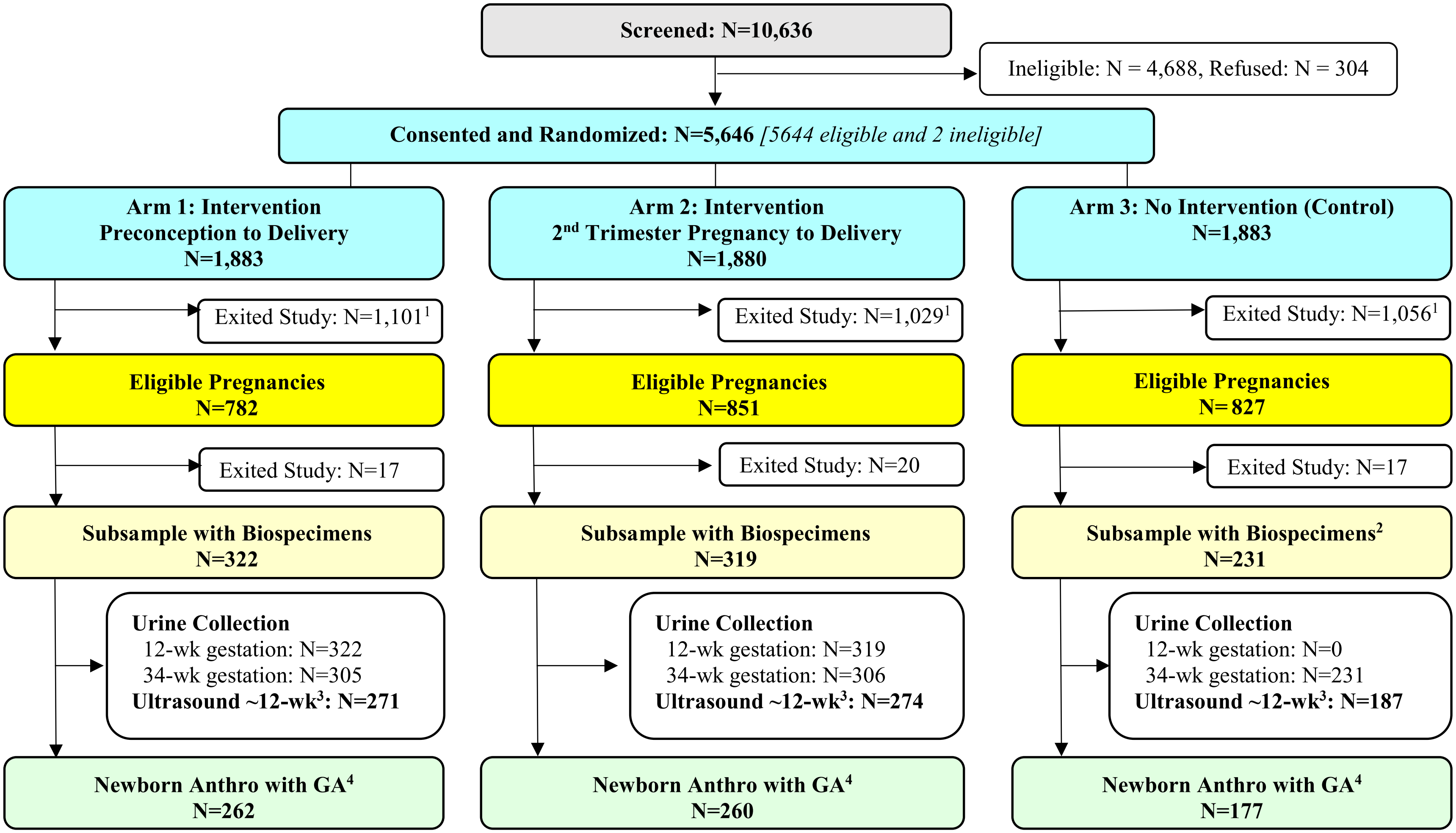

Arm 1 commenced the supplement ≥ 3 months prior to conception and continued through delivery; Arm 2 commenced the same intervention late in the first trimester (after sample collection) and continued until delivery; Arm 3 (Control) received no study supplements.

[1]Primary reason for exit was woman becoming pregnant at < 3 months or not becoming pregnant before enrollment goals reached *(Hambidge KM, et al (2019). A multicountry randomized controlled trial of comprehensive maternal nutrition supplementation initiated before conception: the Women First trial. Am J Clin Nutr, 109(2), 457-469. doi:10.1093/ajcn/nqy228).*

[2]No samples collected for Arm 3 in India.

[3]Gestational age (GA) at birth is defined as the GA determined by ultrasound based on the ultrasound plus time until birth if the ultrasound was done between 6 wk + 0 d to 13 wk + 6 d, and the GA at birth is between 24 wk + 0 d and 42 wk + 6 d. If the ultrasound was not conducted during the GA interval previously mentioned, then the GA at birth is missing.

[4]Newborn anthropometry was obtained for live newborns with three length, weight, and head circumference measurements taken within 48 hours of delivery.
