# Supplementary material for: Improved first trimester maternal iodine status with preconception supplementation: The Women First Trial
Source: Matern Child Nutr. 2021 May 25;17(4):e13204. doi: 10.1111/mcn.13204 (PMC8476419; doi:10.1111/mcn.13204)
Supplement: Supplementary file 2 — Figure S2 Urinary Iodine Concentration (UIC) vs Iodine:Creatinine Ratio (I/Cr) at 12‐ and 34‐wk gestation by Arm [file MCN-17-e13204-s002.pdf]

Supplemental Figure 2. Urinary Iodine Concentration (UIC) vs Iodine:Creatinine Ratio (I/Cr) at 12- and 34-wk gestation by Arm

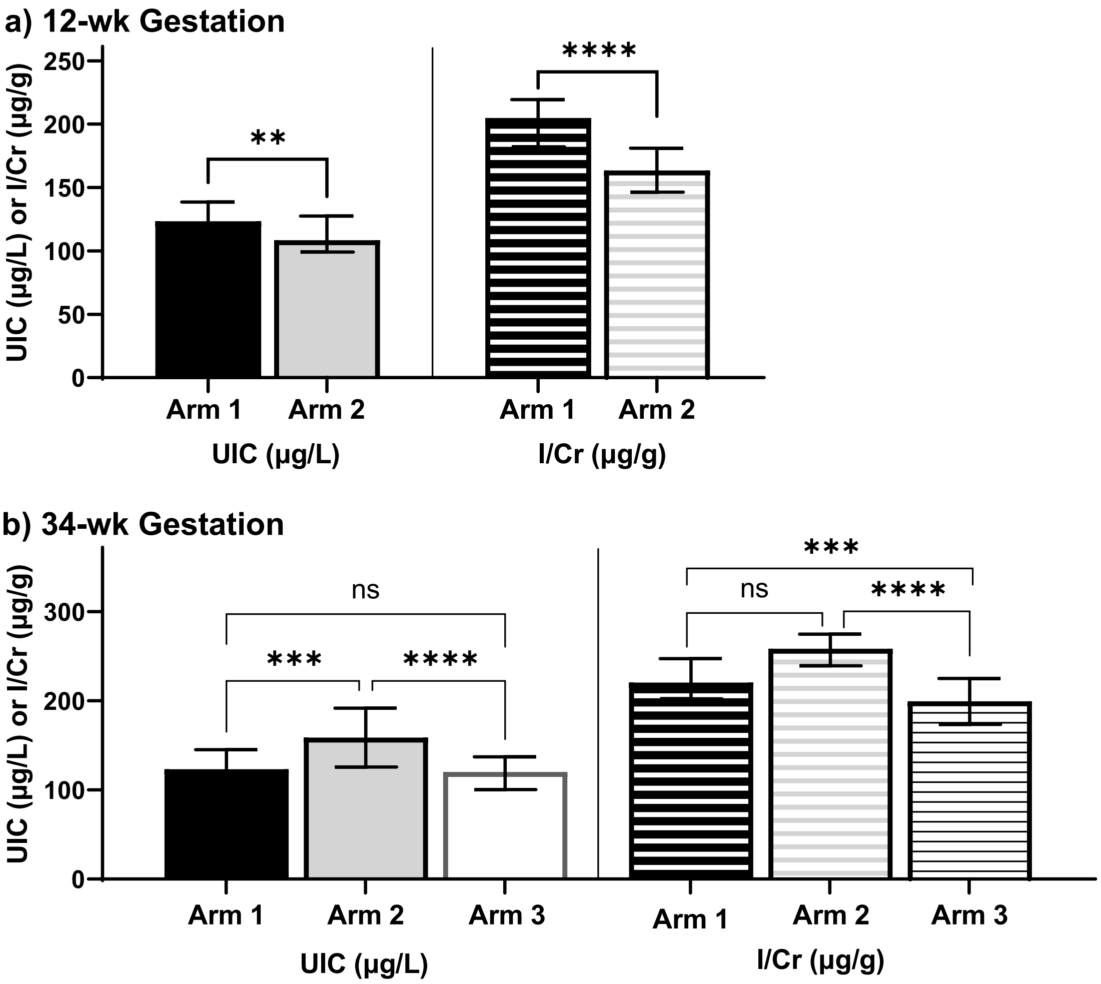

Data presented as median (95% CI). Arm comparisons were done by T-test at 12-wk gestation and ANOVA at 34-wk gestation. **p = 0.0053; ***p < 0.0005; ****p < 0.0001.

Arm 1 commenced the supplement ≥3 months prior to conception and continued through delivery; Arm 2 commenced the same intervention late in the first trimester (after sample collection) and continued until delivery; Arm 3 (Control) received no study supplements. No samples were collected for Arm 3 in India.
