# Supplementary material for: Improved first trimester maternal iodine status with preconception supplementation: The Women First Trial
Source: Matern Child Nutr. 2021 May 25;17(4):e13204. doi: 10.1111/mcn.13204 (PMC8476419; doi:10.1111/mcn.13204)
Supplement: Supplementary file 3 — Figure S3 Iodine to creatinine ratio (I/Cr, μg/g) by categories of maternal status at 34 weeks gestation by site and by arm [file MCN-17-e13204-s006.pdf]

Supplemental Figure 3. Iodine to creatinine ratio (I/Cr, µg/g) by categories of maternal status at 34 weeks gestation by site and by arm

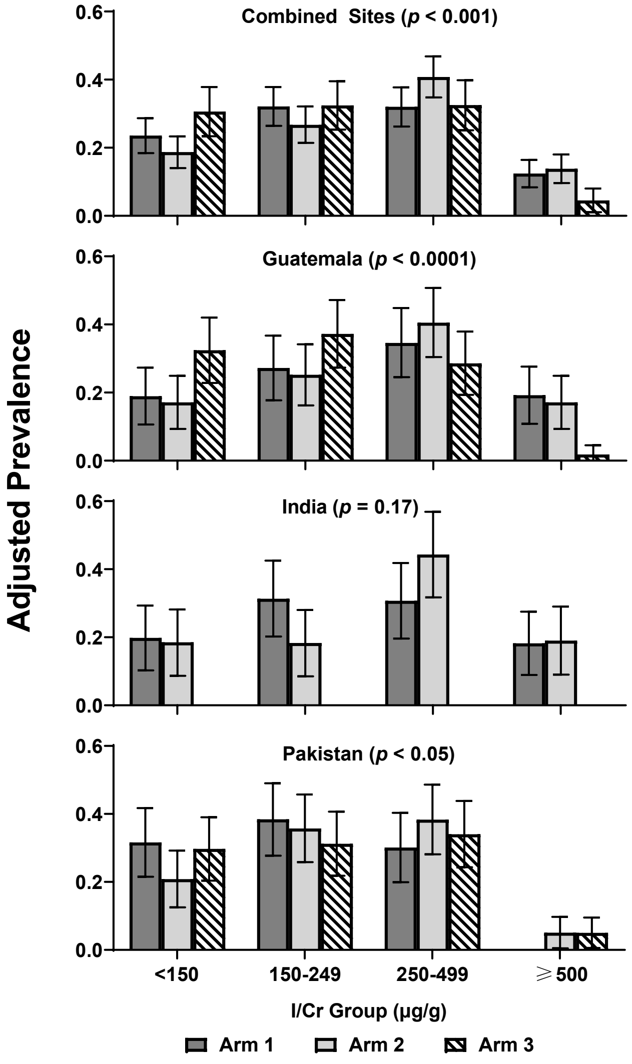

Multinomial logistic regression was used to investigate the relationship between I/Cr category as the outcome and arm. All analyses were adjusted for iodine ≤ 25 µg/L. The combined sites analysis was also adjusted for site. Outliers were removed prior to analysis. Data presented as adjusted mean (95% CI).

Arm 1 commenced the supplement ≥3 months prior to conception and continued through delivery; Arm 2 commenced the same intervention late in the first trimester (after sample collection) and continued until delivery; Arm 3 (Control) received no study supplements. No samples were collected for Arm 3 in India.
