# Supplementary material for: Improved first trimester maternal iodine status with preconception supplementation: The Women First Trial
Source: Matern Child Nutr. 2021 May 25;17(4):e13204. doi: 10.1111/mcn.13204 (PMC8476419; doi:10.1111/mcn.13204)
Supplement: Supplementary file 4 — Figure S4 Categorical iodine to creatinine ratio (I/Cr, μg/g) groups and dichotomous birth outcomes for combined sites at 12 weeks gestation [file MCN-17-e13204-s001.pdf]

Supplemental Figure 4. Categorical iodine to creatinine ratio (I/Cr, µg/g) groups and dichotomous birth outcomes for combined sites at 12 weeks gestation

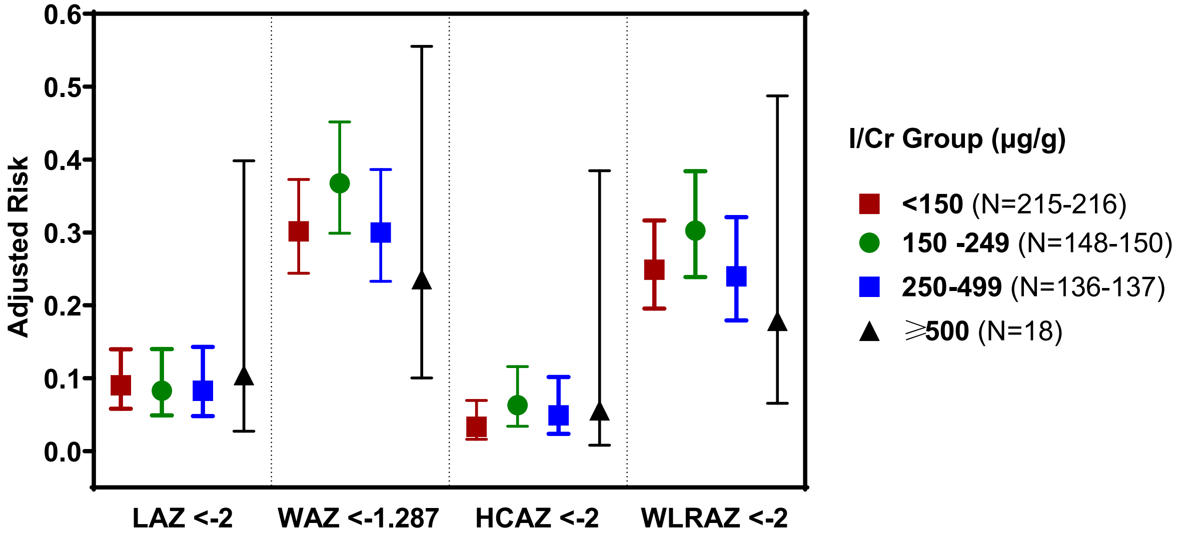

Log-binomial regressions were used to investigate the relationship between dichotomous gestational age adjusted newborn anthropometry measures as the outcome and I/Cr categories as the primary predictor. All models excluded outliers, and included arm and iodine ≤ 25 µg/L as covariates. Outliers were removed prior to analysis. No significant differences between I/Cr groups were found for any of these dichotomous anthropometric outcomes. Data presented as mean (95% CI).

Abbreviations: HCAZ, head circumference-for-age Z-score; LAZ, length-for-age Z-score; LWRAZ, length to weight ratio-for-age Z-score; WAZ, weight-for-age Z-score.
