# Supplementary material for: Improved first trimester maternal iodine status with preconception supplementation: The Women First Trial
Source: Matern Child Nutr. 2021 May 25;17(4):e13204. doi: 10.1111/mcn.13204 (PMC8476419; doi:10.1111/mcn.13204)
Supplement: Supplementary file 5 — Figure S5 Categorical iodine to creatinine ratio (I/Cr, μg/g) groups and continuous birth outcomes at 34 weeks for combined sites [file MCN-17-e13204-s003.pdf]

**Supplemental Figure 5.** Categorical iodine to creatinine ratio (I/Cr, µg/g) groups and continuous birth outcomes at 34 weeks for combined sites

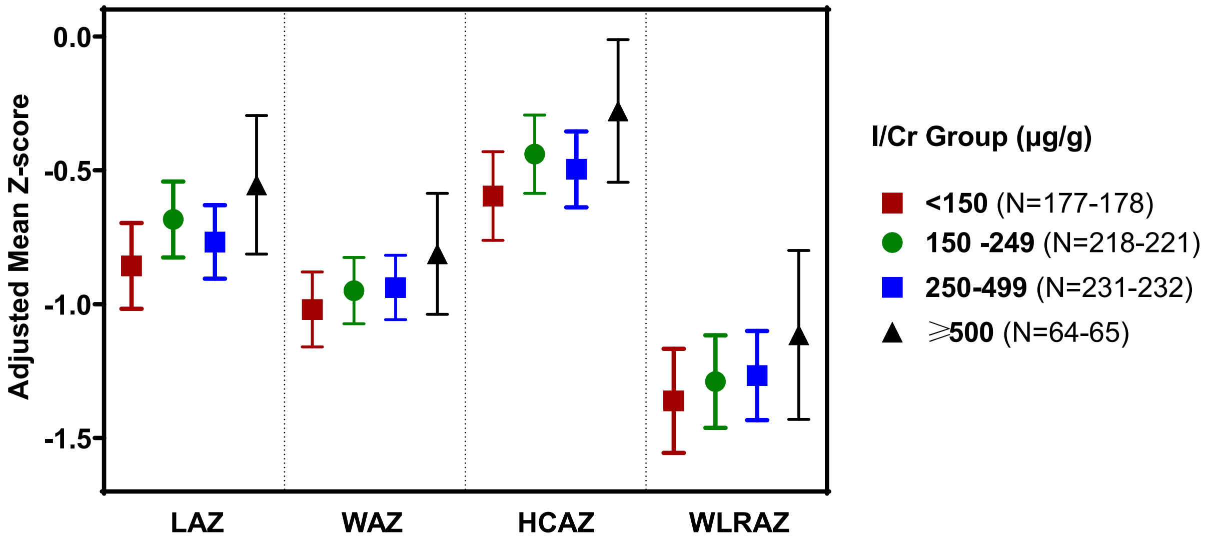


Multiple linear regression models were used with gestational age adjusted continuous newborn anthropometry measures as the outcome and I/Cr categories as the primary predictor and adjusting for arm. Outliers were removed from analyses and low iodine values were adjusted for by including an indicator for iodine ≤ 25 µg/L as a predictor in the linear model. No significant differences between I/Cr groups were found for any of these continuous anthropometric outcomes. Data presented as mean (95% CI).

Abbreviations: HCAZ, head circumference-for-age Z-score; LAZ, length-for-age Z-score; LWRAZ, length to weight ratio-for-age Z-score; WAZ, weight-for-age Z-score.
