# Supplementary material for: Improved first trimester maternal iodine status with preconception supplementation: The Women First Trial
Source: Matern Child Nutr. 2021 May 25;17(4):e13204. doi: 10.1111/mcn.13204 (PMC8476419; doi:10.1111/mcn.13204)
Supplement: Supplementary file 6 — Figure S6 a and b Mediation analyses at 12 weeks [file MCN-17-e13204-s005.pdf]

Supplemental Figure 6a and 6b. Mediation analyses at 12 weeks

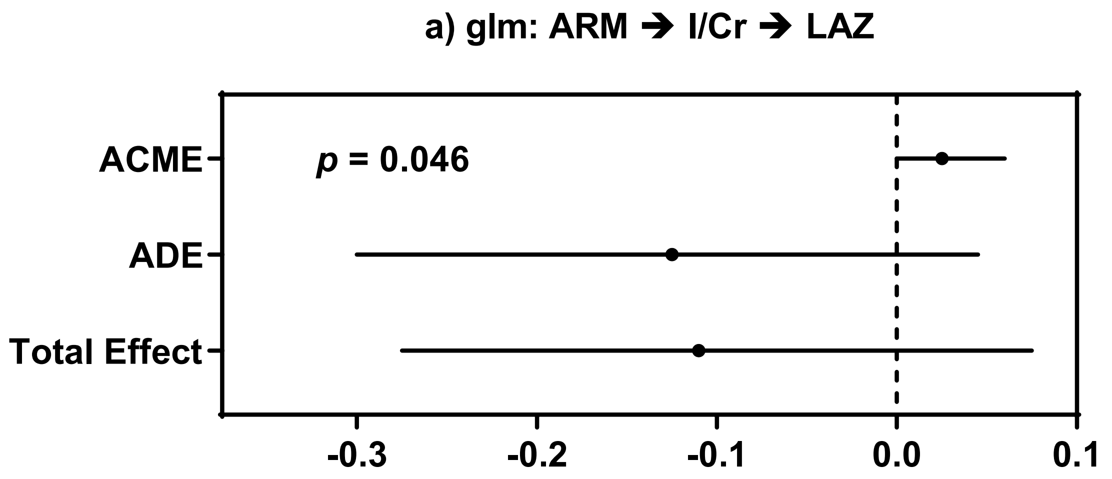

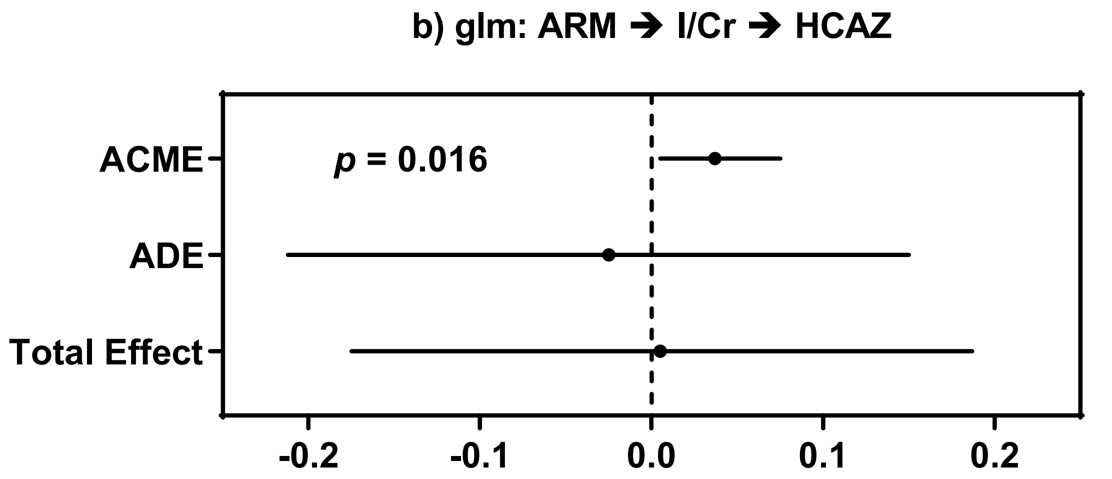

Mediation analysis was performed using the *mediate* function from the mediation R package v4.5.0. The models adjusted for iodine ≤ 25 µg/L and removed outliers. 1000 simulation replicates were used. Data shown represents mean (lower and upper 95% CI).

Abbreviations: ACME, average causal mediation effects; ADE, average direct effects; GLM, general logistical model; HCAZ, head circumference-for-age Z-score; I/Cr, iodine to creatine ratio (µg/g); LAZ, length-for-age Z-score.
